# Prediction of neuromodulatory effect of paired associative stimulation on motor recovery outcomes in spinal cord injury: a pilot animal study

**DOI:** 10.1515/tnsci-2025-0408

**Published:** 2026-11-11

**Authors:** Muhammad Adeel, Bor-Shing Lin, Hung-Chou Chen, Tsung-Hsun Hsieh, Wei-Lun Lo, Chun-Wei Wu, Yu-Ting Li, Kenneth J. Gustafson, Chih-Wei Peng

**Affiliations:** The Master Program in Smart Healthcare Management, International College of Sustainability Innovations, National Taipei University, New Taipei City, Taiwan; Department of Computer Science and Information Engineering, National Taipei University, New Taipei City, Taiwan; Department of Physical Medicine and Rehabilitation, School of Medicine, College of Medicine, Taipei Medical University, Taipei, Taiwan; Department of Physical Medicine and Rehabilitation, Shuang Ho Hospital, Taipei Medical University, New Taipei City, Taiwan; School of Physical Therapy and Graduate Institute of Rehabilitation Science, College of Medicine, Chang Gung University, Taoyuan City, Taiwan; Neuroscience Research Center, Chang Gung Memorial Hospital, Taoyuan City, Taiwan; Department of Surgery, Division of Neurosurgery, Shuang Ho Hospital, Taipei Medical University, New Taipei City, Taiwan; Department of Surgery, School of Medicine, College of Medicine, Taipei Medical University, Taipei, Taiwan; School of Biomedical Engineering, College of Biomedical Engineering, Taipei Medical University, Taipei, Taiwan; National Center for Instrumentation Research, National Institutes of Applied Research, Hsinchu, Taiwan; Department of Biomedical Engineering, Case School of Engineering School of Medicine, Case Western Reserve University, Cleveland, Ohio, USA; Graduate Institute of Biomedical Optomechatronics, College of Biomedical Engineering, Taipei Medical University, Taipei, Taiwan

**Keywords:** electric stimulation therapy, neurological injury, neurophysiology, rehabilitation medicine

## Abstract

**Objectives:**

Limitation in physical functioning is common after spinal cord injury (SCI). Paired Associative stimulation (PAS) can impact brain and spinal cord networks to restore functionality. Therefore, this study aimed to predict the recovery models using motor evoked potential (MEP) and Basso, Beattie, and Bresnahan (BBB) scores in an animal SCI model after 4 weeks of PAS.

**Methods:**

A total of thirty-three adult Sprague-Dawley rats were recruited and divided into six groups including normal, sham, and four stimulation intervention groups. Four different combinations of PAS were employed for 4 weeks, including (1) 3 min rTMS-iTBS/ts-DCS (2), 3 min rTMS-iTBS/ts-iTBS (3), 20 min rTMS/ts-DCS, and (4) 20 min rTMS/ts-iTBS. The MEP was recorded two times before and six times after stimulation. The BBB score was measured after every week for 4 weeks. The MEP and BBB scores with different combinations were used to compute linear regression models for motor recovery in an SCI animal model.

**Results:**

Post-MEP amplitude was associated with locomotor (BBB) scores in different stimulation groups from week 2 to week 4 (adjusted R^2^=0.77 to 0.99, p=0.001–0.046, with 95 % CIs reported for each model). Post-MEP recordings from all four weeks were closely correlated with BBB scores in all groups each week, often involving post-MEP amplitude at 5 and 10 min. However, timing, testing week, and stimulation group differed across models despite relying on a single marker.

**Conclusions:**

The 3 min rTMS-iTBS/ts-DCS, 3 min rTMS-iTBS/ts-iTBS, 20 min rTMS/ts-DCS, and 20 min rTMS/ts-iTBS models predicted significant outcomes during week 2, week 3, and week 4 for locomotor recovery (BBB scores) using post-MEP after paired stimulations in incomplete SCI rats. Because each stimulation group had a small sample size (n=3–5) and the study tested a variety of predictor variables, the findings are best regarded as preliminary and exploratory, generating hypotheses about associations rather than validating predictors of recovery.

## Introduction

Spinal cord injury (SCI) causes serious abnormality and lifelong disability. Many people are affected by SCI every year, and about 90 % of injuries are due to traumatic incidents [1]. The neuronal damage after SCI results in various impairments, including sensory and motor dysfunction, aberrant reflexes, and autonomic problems [2], 3]. The long-term consequences of SCI not only affect the quality of life of individuals but also exert immense economic pressure for rehabilitation [4], 5]. Hence, recovery-based treatment options should be necessary to translate into the clinical setting. Paired associative stimulation (PAS) in the form of simultaneous brain and spinal cord stimulation is the latest non-invasive neuromodulation technique with very less side effects in the SCI population. It utilizes repetitive transcranial magnetic stimulation (rTMS) and transspinal-direct current stimulation (ts-DCS). These stimulations affect neuroplastic mechanisms of pre- and postsynaptic neuronal activation more effectively in the SCI population than other commonly used interventions like physiotherapy, occupational therapy, and electrical stimulation [6], 7]. PAS regulates cortical excitability to induce neuroplasticity which can enhance motor and memory functions in patients [8], [9], [10], [11], [12], [13], [14].

Among animals, the rat is the primary model to study SCI and treatment strategies [15], [16], [17]. Much of the pathological events following SCI are investigated through rat models. Thus, it is the key choice for experimental research on SCI [18]. Marufa et al. reported the effects of intermittent theta burst stimulation (iTBS) on locomotor function, motor plasticity, and axonal regeneration in an incomplete SCI rat model. They have observed an increase in motor plasticity and axonal regeneration through GAP-43 expression [19]. A significant change in motor evoked potential (MEP) and Basso, Beattie, and Bresnahan (BBB) scores was also reported in an animal SCI model after PAS [20]. Another research evaluated the safety of short- and long-term PAS on brain biomarkers and scalp tissue in Sprague-Dawley rats and reported no adverse effect after tDCS-iTBS stimulations [8]. Among the clinical population, PAS was tested in incomplete SCI survivors with enhanced MEP and a significant increase in lower extremity motor scale (LEMS) score [6]. Another study reported the combined effect of tDCS and iTBS in chronic stroke survivors which enhanced their upper limb motor functions [21].

To forecast the outcome of motor functions after traumatic SCI is crucial to implement management approaches that may reduce the cost and provide a better outlook of long-term recovery goals. For this instance, Kaminski et al. constructed a linear regression model for Spinal Cord Independence Measure (SCIM) after 1 year of SCI trauma. The predictors of functional outcome were measured during the acute hospital stay including age, sex, level and type of injury, comorbidities, American Spinal Injury Association (ASIA) Impairment Scale (AIS), ASIA Motor Score (AMS), ASIA Light Touch (LT) score, ASIA Pin Prick (PP) score, Injury Severity Score (ISS), Traumatic Brain Injury (TBI), and delay from trauma to surgery. Only four variables for SCIM at 1 year including LT, AIS grade, ISS, and AMS were chosen in the model with an R^2^ of 0.57 [22]. Another study predicted functional recovery in chronic cervical SCI using bootstrapping analysis and multivariate logistic regression using predictors such as age, gender, the motor and sensory cumulative scores of the International Standards for Neurological Classification of Spinal Cord Injury (ISNCSCI), and neurophysiological variables (motor evoked potentials, sensory evoked potentials, and nerve conduction study). The significant models with the area under the curve (AUC) value 0.956 (95 % CI: 0.930–0.982; p=0.019*) were obtained [23].

To the best of our knowledge, very few research studies are available to predict the long-term recovery in the SCI population. Hence, our current study is the first to use MEP and BBB scores after PAS to predict recovery outcomes of motor functions for long-term care in an animal SCI model. In this aspect, this prediction of recovery in an animal SCI model is expected to be translated to the clinical population. In our previous studies, we explored the effects of rTMS and iTBS on the MEP change in chronic SCI, chronic stroke survivors, and animal SCI model [6], 8], [19], [20], [21]. Two of them reported the efficacy of brain and spinal cord stimulations with an increased MEP amplitude and improved BBB score in an animal SCI model [20] and improvements in MEP amplitude, MEP latency, LEMS, and MAS (Modified Ashworth Scale) scores in SCI survivors [6]. Therefore, we aimed to predict the motor recovery outcomes in an animal SCI model after PAS using MEP amplitude and BBB scores with multi-linear regression modeling as an exploratory study into two different categories. The results obtained from the study may help clinicians to predict long-term motor recovery after SCI to implicate in the clinical setting.

## Materials and methods

### Animal handling and SCI surgery

This study was reviewed and approved by [Taipei Medical University’s Institutional Animal Care and Use Committee], with the approval number: [IACUC-TMU, LAC-2018-0402]. We included thirty-three male adult Sprague Dawley rats from BioLASCO, and their body weights varied from 300 to 450 g. The nine rats were excluded because they died due to infections and the rest of the twenty-four were arranged into six groups: Group (1) normal: intact rats, Group (2) sham: spinal cord injured rats, Group (3) rTMS-iTBS/ts-DCS: spinal cord injured rats with transcranial iTBS and transspinal DCS, Group (4) rTMS-iTBS/ts-iTBS: spinal cord injured rats with transcranial iTBS and electrically transspinal iTBS, Group (5) rTMS/ts-DCS: spinal cord injured rats with transcranial rTMS and transspinal DCS, and (6) rTMS/ts-iTBS: spinal cord injured rats with transcranial rTMS and electrically transspinal iTBS, respectively. All of them were taken care of in a hygienic, climate- and moisture-controlled animal facility according to the ethical standards and acclimatized for 7–10 days. They were placed in a 12-h light/12-h dark cycle and provided with continuous pellet food and water *ad libitum*.

The surgical procedure to raise an animal SCI model was explained in our previous studies [19], 20]. An intraperitoneal (i.p.) injection of tiletamine-zolazepam (50 mg/kg, i.p.; zoletil, Vibac, Carros, France) and xylazine (10 mg/kg, rompun, Bayer, Leverkusen, Germany) was given to perform dorsal laminectomy between T9 (thoracic) and T11 which exposed the T10 spinal cord. A SCI was induced through a compression clamp applied for 60 s. During the surgery, an aseptic environment was maintained and postoperative care with daily check-ups and bladder voiding was performed two times a day. To avoid infections, an intramuscular injection of ampicillin (100 mg/kg, subcutaneously (s.c.), Novopharm, Toronto, Canada) was injected for five days after the surgery [19], 24].

### Experimental intervention

Each animal was stationed in a stereotaxic apparatus following i.p. anesthesia with tiletamine‐zolazepam (50 mg/kg, i.p.; zoletil) and xylazine (10 mg/kg, rompun) [25]. A monopolar uninsulated [27] G stainless steel needle electrodes (Axon Systems, Hauppauge, NY, USA) were inserted into the belly of the biceps femoris of the hindlimb muscle. The tail was used for the ground electrode while the reference electrode was implanted in a paw [26]. The MEP signal was recorded through a Biopac MP-36R four-channel device (Biopac System, Goleta, CA, USA), and the MEP stimulations were applied with a MagVenture MagPro and Cool-40 Rat circular coil (Tonica Electronic, Farum, Denmark). To evoke hindlimb MEPs, the circular coil was placed in slight contact with the scalp surface (locator coordinates AP: ±1.0 mm; ML: ±1.25 mm; bregma the center) [27]. A single TMS pulse evoked MEP in the SCI model to determine cortical excitability in each session. The resting motor threshold (RMT) is the minimum intensity of TMS which evokes MEP from the biceps femoris at 20 µV amplitude in five continuous stimulations of relaxed muscle under anesthesia [26]. The intensity of TMS for RMT was 100 % of the machine output, reflecting the greatest magnetic field strength [26]. The MEP was recorded twice before the intervention and six times after the intervention every 5 min. The recorded MEP is composed of 15 single pulses at a 10 s gap with an RMT of 120 %. Peak-to-peak MEP amplitude was processed offline [19] (Figure 1).

**Figure 1:**
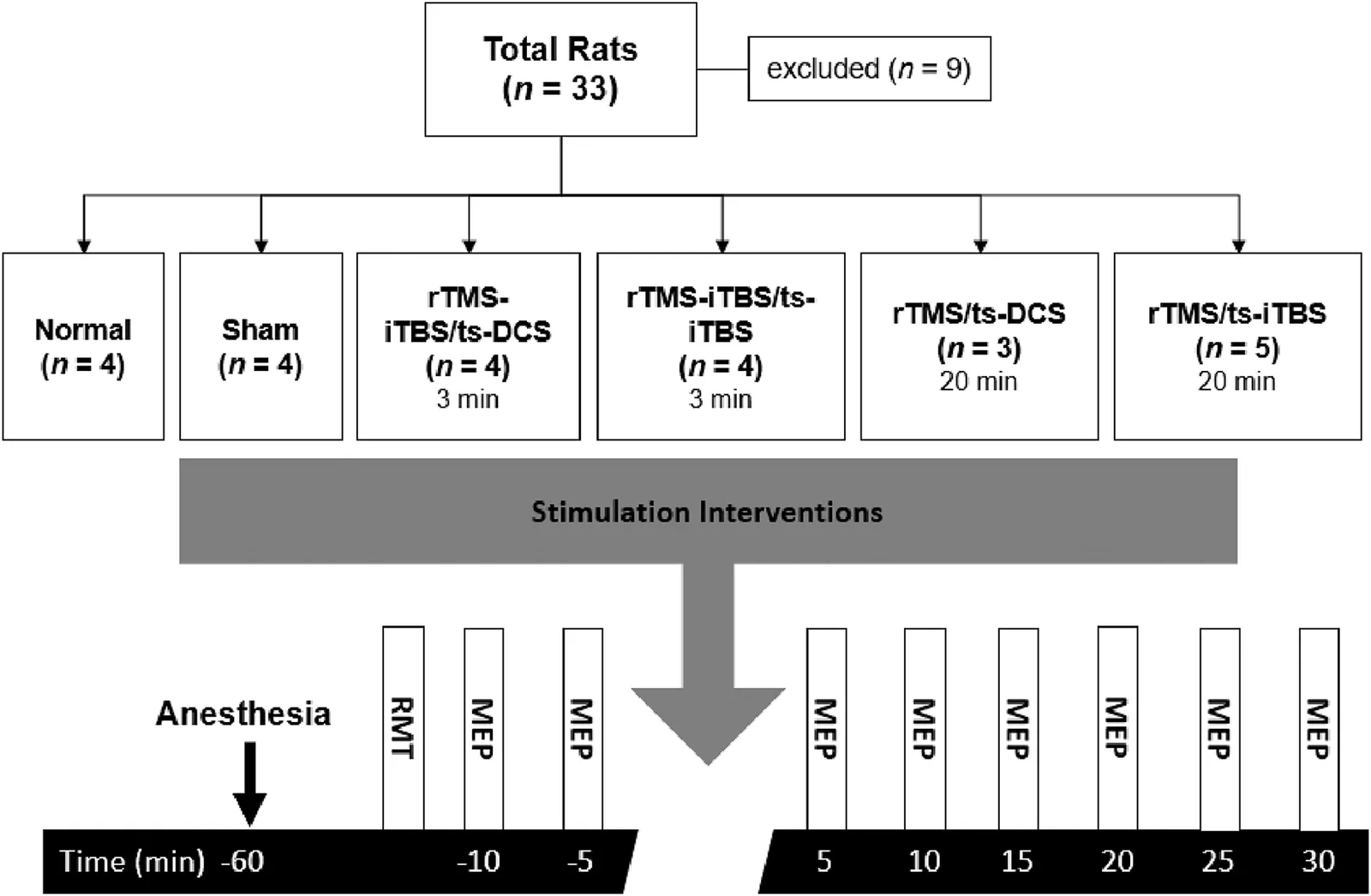
Animal recruitment, MEP assessment, and stimulation interventions for T10 SCI. n, the number of animals; MEP, motor evoked potential; iTBS, intermittent theta burst stimulation; ts-DCS, transspinal-direct current stimulation; ts-iTBS, transspinal-intermittent theta burst stimulation; rTMS, repetitive transcranial magnetic stimulation.

The stimulation interventions for four groups are Group 3: 3 min rTMS-iTBS/ts-DCS: repetitive transcranial magnetic stimulation-intermittent theta burst stimulation (5 Hz)/transspinal-direct current stimulation (0.5 mA), Group 4: 3 min rTMS-iTBS/ts-iTBS: repetitive transcranial magnetic stimulation-intermittent theta burst stimulation (5 Hz)/transspinal-intermittent theta burst stimulation (0.5 mA), Group 5: 20 min rTMS/ts-DCS: repetitive transcranial magnetic stimulation (20 Hz)/transspinal-direct current stimulation (0.5 mA), and Group 6: 20 min rTMS/ts-iTBS: repetitive transcranial magnetic stimulation (20 Hz)/transspinal-intermittent theta burst stimulation (0.5 mA).

Groups (3) and (4) received stimulation intervention for 3 min, 3 times a week for 4 weeks while groups 5 and 6 received 20 mins of stimulation once a week for 4 weeks. The details of each group intervention protocol were explained in the previous study [20] and summarized in Figure 1. The sham group received the same intervention as the other intervention groups, but the coil was inverted above the animal’s head [28]. All of the animals in each group did wheel training for 20 min after the stimulation intervention. The BBB scale, which is used to assess the locomotion of rat hindlimb, consists of 22 points (normal score, 21; no hindlimb function, 0) and is divided into early (0–7), intermediate (8–14), and late (15–21) stages [27]. Each animal was placed in an adjustable open area to record the hindlimb function. The subjectivity of the BBB scale was reduced by using a screening sheet developed by Basso [17], 24], 25], 29].

### Data collection and analysis

The data were analyzed in SPSS (v.17, SPSS, Inc., Chicago, IL, USA) with the significance level p<0.050* for exploratory analysis. The included variables were MEP amplitude and BBB score, as shown in Table S1. We performed univariate analysis using central tendency, dispersion, and frequency of variables. The normal distribution of these variables was evaluated through the Kolmogorov-Smirnov test. The statistical modeling of MEP and BBB scores to predict SCI recovery was conducted using linear regression with a stepwise method, which met the conditions of independence of errors. Collinearity was assessed through variance inflation factors (VIF). The cut-off for VIF is 2.5, and higher values represent the negative correlation [30]. Candidate predictors were restricted *a priori* to [X] and [Y], based on their established relationship to corticospinal integrity and motor recovery. A total of 20 animals from six groups (Group (2) sham, Group (3) rTMS-iTBS/ts-DCS, Group (4) rTMS-iTBS/ts-iTBS, Group (5) rTMS/ts-DCS, and Group (6) rTMS/ts-iTBS, except Group (1) normal were included for model computation (Figure 1).

Two categories of regression models were computed as (1) BBB_w1 a dependent and Post_MEP_w1 (5min, 10min, 15min, 20min, 25min, and 30min) as independent (predictor) variables, the rest of the weeks BBB score and Post_MEP were also tested week vise and (2) BBB_w1 as dependent and Post_MEP_w1,w2,w3,w4 (5 min, 10 min, 15 min, 20 min, 25 min, and 30 min) as independent (predictor) variables, BBB week 2, week 3, and week 4 were also tested individually with all 4 weeks Post_MEP at 5 min, 10 min, 15 min, 20 min, 25 min, and 30 min (Figure 2).

**Figure 2:**
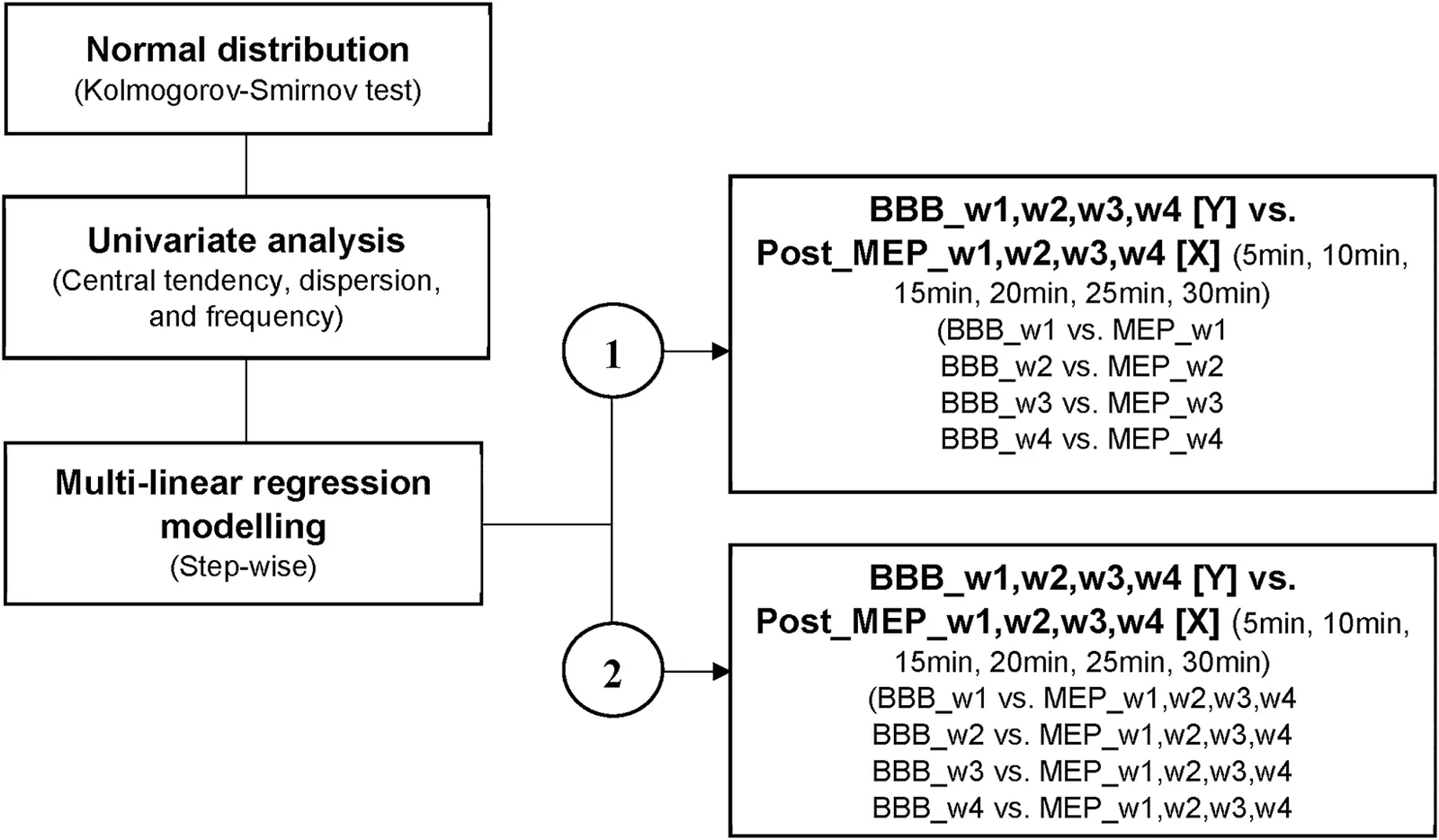
Data analysis and model computation. Post_MEP_w1,w2,w3,w4, MEP after stimulation intervention for each week 1, 2, 3, and 4; BBB_w1_w2,w3,w4, BBB score after week 1, 2, 3, and 4; the variable in each type of model on the left side is a dependent variable [Y] while on the right side is an independent or predictor variable [X] for the linear regression. 1: Method computed models by inputting each week’s BBB score with each respective week’s Post_MEP at 5 min, 10 min, 15 min, 20 min, 25 min, and 30 min. 2: Method computed models by inputting each week’s BBB score with all 4 weeks’ Post_MEP at 5 min, 10 min, 15 min, 20 min, 25 min, and 30 min.

## Results

### Univariate analysis

Among the intevention groups, the BBB score and Pre_MEP for SCI rats reached the highest value for the 20 min rTMS/ts-DCS group (mean=14.20, median=15.00, range=11.20) and (mean=1.10, median=1.10, range=0.29), respectively and Post_MEP attained the highest value for the 3 min rTMS-iTBS/ts-DCS (mean=1.67, median=1.47, range=1.53). (Table S2).

### Linear regression models

#### BBB vs. Post_MEP models for each week (method 1)

Table 1 summarizes the regression models computed for weeks 1–4. We could not estimate a model for week 1 because the BBB score at that time point (BBB_w1) did not add to the model. From week 2 onward, single post-MEP time points were associated with BBB score in several stimulation groups, though the specific time point and group varied from week to week rather than converging on a consistent relationship. Week 2 shows an association with the BBB score for the 3 min rTMS-iTBS/ts-iTBS at 10 min post-MEP (adjusted R^2^=0.868, 95 % CI [−16.965 ∼ −0.469], p=0.045*, VIF=1.00). Week 3 shows association for the 20 min rTMS/ts-DCS at 25 min post-MEP (adjusted R^2^=0.992, 95 % CI [0.720–6.185], p=0.040*, VIF=1.00) and rTMS/ts-iTBS at 5 min post-MEP (adjusted R^2^=0.809, 95 % CI [3.745–26.310], p=0.024*, VIF=1.00) groups. Week 4 shows association for the 20 min rTMS/ts-DCS at 30 min post-MEP (adjusted R^2^=0.996, 95 % CI [13.254–48.515], p=0.029*, VIF=1.00) and rTMS/ts-iTBS at 5 min post-MEP (adjusted R^2^=0.765, 95 % CI [−90.492 ∼ −7.366], p=0.033*, VIF=1.00) and at 5 and 15 min post-MEP (adjusted R^2^=0.968, 95 % CI [−58.582 ∼ −5.736] & [−18.158 ∼ −0.301], p=0.035* & 0.047*, VIF=1.605) groups. Across weeks, predictors were post-MEP amplitude at 5, 10, 15, 25, and 30 min: week 2 and week 3 used 5-, 10-, and 25-min measurements, while week 4 models used 5-, 15-, and 30-min measurements.

**Table 1:** Method 1: multi-linear regression models between BBB [Y] and Post_MEP [X] in SCI rats. (n=20).

	Group	Model	R	R^2^ adjusted	Beta	Std. error	95 % CIs Lower – higher	Sig	Collinearity statistics	
									Tolerance	VIF
Week 2	**rTMS-iTBS/ts-iTBS (3 min)**	Constant	*0.955*	*0.868*	19.818	3.155	6.242–33.394	0.024		
		Post_10 min_w2			−8.717	1.917	−16.965–−0.469	*0.045* ^*a*^	1.000	*1.000*
Week 3	**rTMS/ts-DCS (20 min)**	Constant	*0.998*	*0.992*	7.344	0.505	0.929–13.758	0.044		
		Post_25 min_w3			3.453	0.215	*0.720*–*6.185*	*0.040* ^*a*^	1.000	*1.000*
	**rTMS/ts-iTBS (20 min)**	Constant	*0.926*	*0.809*	−10.727	4.733	*−25.789*–*4.334*	0.108		
		Post_5 min_w3			15.027	3.545	*3.745*–*26.310*	*0.024* ^*a*^	1.000	*1.000*
Week 4	**rTMS/ts-DCS (20 min)**	Constant	*0.999*	*0.996*	−12.330	1.212	*−27.730*–*3.070*	0.062		
		Post_30 min_w4			30.885	1.388	*13.254*–*48.515*	*0.029* ^*a*^	1.000	*1.000*
	**rTMS/ts-iTBS (20 min)**	Constant	*0.908*	*0.765*	51.433	11.169	*15.889*–*86.977*	0.019		
		Post_5 min_w4			−48.929	13.060	*−90.492*–*−7.366*	*0.033* ^*a*^	1.000	*1.000*
		Constant	*0.992*	*0.968*	46.439	4.294	*27.962*–*64.917*	0.008		
		Post_5 min_w4			−32.159	6.141	*−58.582*–*−5.736*	*0.035* ^*a*^	0.623	*1.605*
		Post_15 min_w4			−9.229	2.075	*−18.158*–*−0.301*	*0.047* ^*a*^	0.623	*1.605*

BBB scores for weeks 1, 2, 3, and 4 were taken as dependent variables, while Post_5, 10, 15, 20, 25, and 30 min MEP_w1,w2,w3,w4 were taken as independent (predictor) variables. Each week’s BBB score is correlated with each respective week’s Post_MEP at 5 min, 10 min, 15 min, 20 min, 25 min, 30 min (Method 1 refers to Figure 2). Week 1 models cannot be computed because the dependent variable BBB_w1 was not added to the model. ^a^Significant p-value < 0.050. Bold text shows different intervention groups; italic text shows higher correlation, significant p-values, and VIF values.

#### BBB vs. Post_MEP 4 weeks models (method 2)

Table 2 further explores the full study intervention period, testing each week’s BBB score with post-MEP recordings for all four-week sessions (5 through 30 min post-stimulation) to identify the strongest post-MEP predictor for locomotor recovery after stimulation interventions. The results showed significant models for each week in nearly every group, with adjusted R^2^ values spanning from 0.786 to 0.998 and predictor p-values ranging from 0.001 to 0.046. Variance inflation factors remained at or near 1.00 across all models, rising only to 1.036 in the single two-predictor model for week 1.

**Table 2:** Method 2: multi-linear regression models between BBB [Y] and Post_MEP in SCI rats. (n=20).

	Group	Model	R	R^2^ adjusted	Beta	Std. error	95 % CIs Lower – higher	Sig	Collinearity statistics	
									Tolerance	VIF
**Week 1**	**rTMS-iTBS/ts-iTBS (3 min)**	Constant	*0.954*	*0.866*	23.378	4.200	5.305–41.451	0.031		
		Post_25** **min_w4			−21.380	4.742	−41.785–−0.976	0.046^a^	1.000	1.000
	**rTMS/ts-DCS (20 min)**	Constant	*0.999*	*0.998*	−1.721	0.489	−7.931–4.490	0.176		
		Post_5** **min_w3			6.879	0.243	3.790–9.967	0.022^a^	1.000	1.000
	**rTMS/ts-iTBS (20 min)**	Constant	*0.916*	*0.786*	−21.381	6.306	−41.450–−1.313	0.043		
		Post_5** **min_w3			18.725	4.724	3.692–33.758	0.029^a^	1.000	1.000
		Constant	*0.999*	*0.995*	−16.860	1.048	−21.371–−12.350	0.004		
		Post_5** **min_w3			20.255	0.738	17.082–23.428	0.001^b^	0.966	1.036
		Post_30** **min_w3			−4.920	0.439	−6.810–−3.030	0.008^a^	0.966	1.036
**Week 2**	**rTMS-iTBS/ts-iTBS (3 min)**	Constant	*0.960*	*0.883*	25.954	4.121	8.223–43.685	0.024		
		Post_25** **min_w4			−22.659	4.653	−42.677–−2.641	0.040^a^	1.000	1.000
	**Sham**	Constant	*0.989*	*0.967*	49.697	4.301	31.192–68.202	0.007		
		Post_25** **min_w3			−31.847	3.401	−46.481–−17.214	0.011^a^	1.000	1.000
	**rTMS/ts-iTBS (20 min)**	Constant	*0.955*	*0.882*	35.932	5.132	19.600–52.265	0.006		
		Post_10** **min_w4			−32.257	5.803	−50.725–−13.788	0.011^a^	1.000	1.000
**Week 3**	**rTMS-iTBS/ts-iTBS (3 min)**	Constant	*0.992*	*0.977*	23.502	1.413	17.421–29.583	0.004		
		Post_10** **min_w2			−9.683	0.859	−13.377–−5.988	0.008^a^	1.000	1.000
	**Sham**	Constant	*0.979*	*0.939*	−1.687	1.910	−9.906–6.532	0.470		
		Post_5** **min_w1			12.278	1.790	4.575–19.981	0.021^a^	1.000	1.000
	**rTMS/ts-DCS (20 min)**	Constant	*0.999*	*0.996*	−12.330	1.212	−27.730–3.070	0.062		
		Post_30** **min_w4			30.885	1.388	13.254–48.515	0.029^a^	1.000	1.000
	**rTMS/ts-iTBS (20 min)**	Constant	*0.926*	*0.809*	−10.727	4.733	−25.789–4.334	0.108		
		Post_5** **min_w3			15.027	3.545	3.745–26.310	0.024^a^	1.000	1.000
**Week 4**	**rTMS-iTBS/ts-iTBS (3 min)**	(Constant)	*0.989*	*0.967*	23.729	1.593	16.876–30.581	0.004		
		Post_10** **min_w2			−9.162	0.968	−13.325–−4.999	0.011^a^	1.000	1.000
	**rTMS/ts-DCS (20 min)**	(Constant)	*0.999*	*0.996*	−12.330	1.212	−27.730–3.070	0.062		
		Post_30** **min_w4			30.885	1.388	13.254–48.515	0.029^a^	1.000	1.000
	**rTMS/ts-iTBS (20 min)**	(Constant)	*0.919*	*0.792*	−13.133	5.782	−31.534–5.268	0.108		
		Post_5** **min_w3			17.469	4.331	3.686–31.253	0.027^a^	1.000	1.000

BBB scores for weeks 1, 2, 3, and 4 were taken as dependent variables, while Post_5, 10, 15, 20, 25, and 30min MEP_w1,w2,w3,w4 were taken as independent (predictor) variables. Each week’s BBB was regressed against post-MEP from all four weeks (w1–w4) at MEP at 5** **min, 10** **min, 15** **min, 20** **min, 25** **min, and 30** **min (Method 2 refers to Figure 2). ^a^Significant p-value < 0.050; ^b^significant p-value < 0.001. Bold text shows different intervention groups; italic text shows higher correlation, significant p-values, and VIF values.

Throughout the four-week stimulation intervention, post-MEP amplitude at 5 and 10 min was a frequent predictor, observed across multiple groups and weeks. In contrast, post-MEP amplitude at 25 and 30 min appeared less often across groups. The week of the retained post-MEP recording did not always align with the week of the associated BBB score: many models linked a specific week’s BBB score to post-MEP amplitude recorded in an earlier, the same, or a later testing session, rather than using that week’s concurrent recording. This pattern indicates that corticospinal activity in response to paired stimulation, captured across different weeks, tracked with locomotor status across multiple time points rather than being confined to a single fixed post-stimulation period.

## Discussion

In this study, we used two outcome measures (1) MEP amplitude and (2) BBB score from the animal SCI model after applying PAS for four weeks. The group-wise comparison for each 3 and 20 min stimulation intervention was reported in our previous study [20]. However, in this study, we correlated MEP and BBB scores after PAS to forecast long-term recovery in an animal SCI model. We used different combinations to compute linear regression models, including (Method 1) BBB_w1 vs. Post_MEP_w1, BBB_w2 vs. Post_MEP_w2, and so on, and (Method 2) BBB_w1 vs. Post_MEP_w1,w2,w3,w4, BBB_w2 vs. Post_MEP_w1,w2,w3,w4, and so on (Figure 2).

Based on the results of this study, the models were evaluated for the groups and time factors into two categories. The ***category 1 models in*** Table 1 represented highly correlated significant models for week 2, week 3, and week 4 for 3 min rTMS-iTBS/ts-iTBS, 20 min rTMS/ts-DCS, and 20 min rTMS/ts-iTBS groups. The predictors are Post_5, 10, 15, 25, and 30min MEPs, which have a correlation with the respective week 2, week 3, and week 4 BBB scores. Week 2 predicted model only for 3 min paired stimulation (rTMS-iTBS/ts-iTBS), while week 3 and week 4 predicted models for both 20 min paired stimulations (rTMS/ts-DCS & rTMS/ts-iTBS). The ***category 2 models in*** Table 2 computed significant models across all four weeks. The 3 min rTMS-iTBS/ts-iTBS contributed to significant associations for all four weeks at 10 and 25 min post-MEP; 20 min rTMS/ts-DCS contributed for week 1, week 3, and week 4 at 5 and 30 min post-MEP; and 20 min rTMS/ts-iTBS contributed for week 1, week 2, and week 3 at 5, 10 and 30 min post-MEP, while 3 min rTMS/ts-iTBS contributed only for week 4 at 5 min post-MEP.

The sham group’s BBB score was 11.58 (1.72), higher than that of the other groups except the 20 min rTMS/ts-DCS group, which had a score of 14.20 (5.64). However, the difference in the scores is not significant, making it difficult to explain why this difference occurred. Possible reasons may include random effects, small sample size, or other factors. Additionally, the differences in BBB scores between the sham and other intervention groups are not substantial and vary by a small margin.

One study predicted SCI survivors’ functional recovery at 1 year using ASIA evaluation (AIS grade, LT, and AMS) and the ISS predictors, and obtained an R^2^=0.57 [22]. In our current study, we obtained R^2^ ranging from 0.77 to 0.99. It represents that the physiological parameter MEP and functional recovery BBB score have a strong correlation in predicting long-term recovery in the SCI model after PAS intervention [19], 20]. Another study utilized non-linear regression modeling (XGBoost) to predict functional recovery after SCI (t=6.57, p<0.001) with the following predictors (1) clinical characteristics, including age, American Spinal Injury Association (ASIA) Impairment Scale (AIS) at admission, level of injury and baseline ASIA Motor Score (AMS) (2), MR imaging, mainly Brain and Spinal Injury Center (BASIC) score, and (3) surgical timing, specifically comparing whether surgical decompression was received within 24 h or not [31].

For SCI rehabilitation, TMS can regulate the imbalance between excitability and inhibition of cortical and corticospinal tract networks [32]. In previous studies, dual tDCS through cathodal stimulation inhibits the unaffected lateral hemisphere of stroke patients. In comparison, anodal stimulation increases the affected hemisphere’s excitability by increasing MEP [33]. tDCS has been shown to alleviate spasticity and neuropathic pain caused by stroke, multiple sclerosis, SCI, and other neurological diseases; besides, it leads to somewhat improved patient motor function [34], 35]. Improved MEP latency, MEP amplitude, and lower limb muscle strength were observed with the use of the two PAS protocols (20 Hz-rTMS/tsDCS and rTMS-iTBS/tsDCS) in chronic SCI survivors [6]. Another study [20] reported the change in MEP and BBB scores in an animal SCI model after using PAS for four weeks. The effect of tDCS and iTBS in chronic stroke confirmed the improvement in upper limb hand function assessed through Fugl-Meyer Assessment-Upper Extremity (FMA-UE), Jebsen-Taylor Hand Function Test (JTT), and Finger-to-Nose Test (FNT) [21]. Hence, the application of TMS in the clinical setting can be implemented in the future after large-sample clinical trials because of its effects on motor recovery in chronic SCI and stroke survivors.

### Strengths and limitations

The strengths of the study are: 1) the study targets a gap in describing the prediction of long-term functional recovery after SCI as one of the major knowledge gaps in the field; the MEP–BBB is a novel and clinically relevant approach, 2) the current study is explicit about the kind of evidence it offers, which tells clinician-scientists exactly how much weight to put on the findings and what a properly powered follow-up study would need to look like, 3) the work is applicable to the clinic, not a hypothetical one. The electrophysiological relationship under study, MEP responsiveness as an early marker of later motor recovery, already underlies clinical prediction algorithms used in human SCI cohorts, and paired associative stimulation has already reached early-phase human trials.

The limitations of the study are 1) the small sample size so more animals will be included for future study for prediction models, 2) we only observed the intervention effect for 4 weeks, but the 8–12 weeks intervention effect should be studied by the future research to confirm the reproducibility of correlation between MEP and BBB score, 3) data augmentation and machine learning or deep learning algorithms should be used for long-term recovery models in SCI, 4) methodological issues were experienced during PAS including the death of rats after SCI injury due to urinary infections or other reasons, response to anesthesia dose after SCI surgery, voluntary movement artifacts on MEP, and gain in weight after SCI surgery may affect the BBB score, 5) lack of model computation with demographic data, 6) lack of model validation due to small sample size, 7) the findings of this study come from a single rodent contusion model, and any extrapolation to the clinical setting should be made cautiously. In humans with incomplete SCI, early lower-limb MEP responsiveness is one of the more consistent electrophysiological predictors of later walking capacity and has already been incorporated into multicenter clinical prediction algorithms, most notably within the European Multicenter Study about Spinal Cord Injury (EMSCI) cohort [36], [37], [38], [39]. That this MEP–outcome relationship holds across species suggests the electrophysiological signal tracked in the present study reflects conserved corticospinal physiology rather than a rodent-specific artifact. On the intervention side, paired associative stimulation protocols conceptually related to the one used here have already progressed from spinal-cord-injured rodents into small proof-of-concept human trials, where they have strengthened corticospinal transmission and restored measurable voluntary control over previously paralyzed muscle in people with chronic incomplete SCI [40]. The present data, collected under the tighter experimental control that an animal model allows, may help identify which post-injury MEP profiles indicate that animals – and, by extension, patients – are most likely to respond to this class of neuromodulation. Interpret these correlations with the same caution applied throughout this exploratory pilot study. Each model was fit within a single stimulation group of three to five animals, and candidate predictors were drawn from twenty-four post-MEP measurements spanning all four weeks, so the specific timing and week retained in each model reflect an exploratory search rather than a pre-specified, confirmatory test. We present this as a testable hypothesis for future biomarker-stratified human trials of paired associative stimulation, not as a conclusion this dataset can establish on its own, 8) most of the research using neuromodulation focuses on basic research, so more clinical studies with large sample sizes and randomized controlled trials should be conducted to advance the treatment of SCI using stimulation. However, it is quite challenging to attain widespread application of these interventions in the short term. The potential safety concerns, affordability, availability, as well as biocompatibility and durability of the electrodes remain obstacles to be dealt with to enhance the accessibility of neuromodulatory techniques in the future [5]. That’s why there is a need for the establishment of appropriate stimulation targets and parameters for enhanced intervention outcomes in SCI.

## Conclusions

It is concluded that 3 min rTMS-iTBS/ts-DCS, 3 min rTMS-iTBS/ts-iTBS, 20 min rTMS/ts-DCS, and 20 min rTMS/ts-iTBS have predicted significant linear regression models for locomotor recovery (BBB scores) using post-MEP after week 2, week 3, and week 4 paired stimulation interventions. Thus, we found a significant correlation between MEP and BBB scores in an animal SCI model and developed predictive recovery models. These results may help neuroscientists predict long-term motor recovery after SCI and have implications for clinical practice. This will also help clinicians and physical therapists design exercise programs and evaluate outcome measures based on the level of motor recovery in SCI survivors. Because each stimulation group had a small sample size (n=3–5) and the study tested a variety of predictor variables, the findings are best regarded as preliminary and exploratory, generating hypotheses about associations rather than validating predictors of recovery.

## Supplementary Material

Supplementary Material

## References

[1] Ashammakhi N, Kim HJ, Ehsanipour A, Bierman RD, Kaarela O, Xue C (2019). Regenerative therapies for spinal cord injury. Tissue Eng B Rev.

[2] Ong B, Wilson JR, Henzel MK (2020). Management of the patient with chronic spinal cord injury. Med Clin.

[3] Ehrmann C, Mahmoudi SM, Prodinger B, Kiekens C, Ertzgaard P (2020). Impact of spasticity on functioning in spinal cord injury: an application of graphical modelling. J Rehabil Med.

[4] Alizadeh A, Dyck SM, Karimi-Abdolrezaee S (2019). Traumatic spinal cord injury: an overview of pathophysiology, models and acute injury mechanisms. Front Neurol.

[5] Tian T, Zhang S, Yang M (2023). Recent progress and challenges in the treatment of spinal cord injury. Protein Cell.

[6] Adeel M, Lai C-H, Lin B-S, Chan WP, Liou J-C, Wu C-W (2022). Effects of paired stimulation with specific waveforms on cortical and spinal plasticity in subjects with a chronic spinal cord injury. J Formos Med Assoc.

[7] Ling YT, Alam M, Zheng YP (2020). Spinal cord injury: lessons about neuroplasticity from paired associative stimulation. Neuroscientist: J Neurobiol Neurol Psychiatr.

[8] Adeel M, Chen C-C, Lin B-S, Chen H-C, Liou J-C, Li Y-T (2022). Safety of special waveform of Transcranial electrical stimulation (TES): in vivo assessment. Int J Mol Sci.

[9] Rossi S, Hallett M, Rossini PM, Pascual-Leone A (2009). Safety, ethical considerations, and application guidelines for the use of transcranial magnetic stimulation in clinical practice and research. Clin Neurophysiol: J Clin Neurophysiol.

[10] Nitsche MA, Paulus W (2000). Excitability changes induced in the human motor cortex by weak transcranial direct current stimulation. J Physiol.

[11] Boroojerdi B, Prager A, Muellbacher W, Cohen LG (2000). Reduction of human visual cortex excitability using 1-Hz transcranial magnetic stimulation. Neurology.

[12] Chen R, Classen J, Gerloff C, Celnik P, Wassermann EM, Hallett M (1997). Depression of motor cortex excitability by low-frequency transcranial magnetic stimulation. Neurology.

[13] Pascual-Leone A, Valls-Solé J, Wassermann EM, Hallett M (1994). Responses to rapid-rate transcranial magnetic stimulation of the human motor cortex. Brain: J Neurol.

[14] Cantello R, Gianelli M, Bettucci D, Civardi C, De Angelis MS, Mutani R (1991). Parkinson’s disease rigidity: magnetic motor evoked potentials in a small hand muscle. Neurology.

[15] Gomes-Osman J, Cortes M, Guest J, Pascual-Leone A (2016). A systematic review of experimental strategies aimed at improving motor function after acute and chronic spinal cord injury. J Neurotrauma.

[16] Reier PJ, Lane MA, Hall ED, Teng YD, Howland DR (2012). Translational spinal cord injury research: preclinical guidelines and challenges. Handb Clin Neurol.

[17] Onifer SM, Rabchevsky AG, Scheff SW (2007). Rat models of traumatic spinal cord injury to assess motor recovery. ILAR J.

[18] Kjell J, Olson L (2016). Rat models of spinal cord injury: from pathology to potential therapies. Dis Model Mech.

[19] Marufa SA, Hsieh T-H, Liou J-C, Chen H-Y, Peng C-W (2021). Neuromodulatory effects of repetitive transcranial magnetic stimulation on neural plasticity and motor functions in rats with an incomplete spinal cord injury: a preliminary study. PLoS One.

[20] Adeel M, Lin B-S, Chen H-C, Lai C-H, Liou J-C, Wu C-W (2022). Motor neuroplastic effects of a novel paired stimulation technology in an incomplete spinal cord injury animal model. Int J Mol Sci.

[21] Chen S-C, Yang L-Y, Adeel M, Lai C-H, Peng C-W (2021). Transcranial electrostimulation with special waveforms enhances upper-limb motor function in patients with chronic stroke: a pilot randomized controlled trial. J NeuroEng Rehabil.

[22] Kaminski L, Cordemans V, Cernat E, M’Bra KI, Mac-Thiong J-M (2017). Functional outcome prediction after traumatic spinal cord injury based on acute clinical factors. J Neurotrauma.

[23] Hupp M, Pavese C, Bachmann LM, Koller R, Group ES, Schubert M (2018). Electrophysiological multimodal assessments improve outcome prediction in traumatic cervical spinal cord injury. J Neurotrauma.

[24] Poon PC, Gupta D, Shoichet MS, Tator CH (2007). Clip compression model is useful for thoracic spinal cord injuries: histologic and functional correlates. Spine.

[25] Rong H, Liu Y, Zhao Z, Feng J, Sun R, Ma Z (2018). Further standardization in the aneurysm clip: the effects of occlusal depth on the outcome of spinal cord injury in rats. Spine.

[26] Rotenberg A, Muller PA, Vahabzadeh-Hagh AM, Navarro X, López-Vales R, Pascual-Leone A (2010). Lateralization of forelimb motor evoked potentials by transcranial magnetic stimulation in rats. Clin Neurophysiol: J Clin Neurophysiol.

[27] Fonoff ET, Dale CS, Pagano RL, Paccola CC, Ballester G, Teixeira MJ (2009). Antinociception induced by epidural motor cortex stimulation in naive conscious rats is mediated by the opioid system. Behav Brain Res.

[28] Aydin-Abidin S, Trippe J, Funke K, Eysel UT, Benali A (2008). High- and low-frequency repetitive transcranial magnetic stimulation differentially activates c-Fos and zif268 protein expression in the rat brain. Exp Brain Res.

[29] Basso DM, Beattie MS, Bresnahan JC (1996). Graded histological and locomotor outcomes after spinal cord contusion using the NYU weight-drop device versus transection. Exp Neurol.

[30] Pardoe I (2020). Applied regression modeling.

[31] Sizheng Z, Boxuan H, Feng X, Dianying Z (2022). A functional outcome prediction model of acute traumatic spinal cord injury based on extreme gradient boost. J Orthop Surg Res.

[32] Benedetti B, Weidenhammer A, Reisinger M, Couillard-Despres S (2022). Spinal cord injury and loss of cortical inhibition. Int J Mol Sci.

[33] Morone G, Capone F, Iosa M, Cruciani A, Paolucci M, Martino Cinnera A (2022). May dual transcranial direct current stimulation enhance the efficacy of robot-assisted therapy for promoting upper limb recovery in chronic stroke?. Neurorehabilit Neural Repair.

[34] Sánchez-Kuhn A, Pérez-Fernández C, Cánovas R, Flores P, Sánchez-Santed F (2017). Transcranial direct current stimulation as a motor neurorehabilitation tool: an empirical review. Biomed Eng Online.

[35] Palm U, Chalah MA, Padberg F, Al-Ani T, Abdellaoui M, Sorel M (2016). Effects of transcranial random noise stimulation (tRNS) on affect, pain and attention in multiple sclerosis. Restor Neurol Neurosci.

[36] Petersen JA, Spiess M, Curt A, Dietz V, Schubert M (2012). Spinal cord injury: one-year evolution of motor-evoked potentials and recovery of leg motor function in 255 patients. Neurorehabilit Neural Repair.

[37] van Middendorp JJ, Hosman AJ, Donders AR, Pouw MH, Ditunno JF, Curt A (2011). A clinical prediction rule for ambulation outcomes after traumatic spinal cord injury: a longitudinal cohort study. Lancet (London, England).

[38] Zörner B, Blanckenhorn WU, Dietz V, Null n, Curt A (2010). Clinical algorithm for improved prediction of ambulation and patient stratification after incomplete spinal cord. Injury.

[39] Curt A, Keck ME, Dietz V (1998). Functional outcome following spinal cord injury: significance of motor-evoked potentials and ASIA scores. Arch Phys Med Rehabil.

[40] Bunday KL, Perez MA (2012). Motor recovery after spinal cord injury enhanced by strengthening corticospinal synaptic transmission. Curr Biol.

