# Supplementary material for: Prediction of neuromodulatory effect of paired associative stimulation on motor recovery outcomes in spinal cord injury: a pilot animal study
Source: Transl Neurosci. 2026 Nov 11;17(1):20250408. doi: 10.1515/tnsci-2025-0408 (PMC13749926; doi:10.1515/tnsci-2025-0408)
Supplement: Supplementary file 1 [file j_tnsci-2025-0408_suppl_001.docx]

| **Table S1:** Outcome variables of the study. | | |
| --- | --- | --- |
| No. | Motor Evoked Potential (MEP) | Basso, Beattie, and Bresnahan (BBB) scale |
| 1. | Pre_10min | Week 0 |
| 2. | Pre_5min | Week 1 |
| 3. | Post_5min | Week 2 |
| 4. | Post_10min | Week 3 |
| 5. | Post_15min | Week 4 |
| 6. | Post_20min | -- |
| 7. | Post_25min | -- |
| 8. | Post_30min | -- |
| Pre_10min, MEP 10min before stimulation intervention; Pre_5min, MEP 5min before stimulation intervention; Post_5min, 10min, 15min, 20min, 25min, 30min, MEP 5, 10, 15, 20, 25, and 30 min after stimulation intervention; Week 0, evaluation one week before inducing SCI surgery; Week 1,2,3,4, evaluation after consecutive weeks of SCI surgery; Each week stimulation intervention before inducing SCI surgery, each week has 8 variables and the total five weeks has 8×5=40 variables; BBB, each week has 1 variable and the total has 5 variables. | | |

**Supplementary Tables**

| **Table S2:** Univariate statistics of SCI rats. (*n* = 20). | | | | | | | | | | |
| --- | --- | --- | --- | --- | --- | --- | --- | --- | --- | --- |
| **Groups** | **BBB** | | | **Pre_MEP** | | | **Post_MEP** | | | |
|  | **Mean (SD)** | **Median** | **Min~Max** | **Mean (SD)** | **Median** | **Min~Max** | **Mean (SD)** | | **Median** | **Min~Max** |
| **Control** | 20.6 (0.80) | 21.00 | 19.40~21.00 | 1.08 (0.08) | 1.10 | 0.97~1.15 | 1.30 (0.67) | 1.00 | | 0.91~2.31 |
| **Sham** | 11.58 (1.72) | 11.90 | 9.20~13.30 | 1.10 (0.07) | 1.11 | 1.01~1.18 | 1.61 (0.62) | 1.34 | | 1.22~2.52 |
| **rTMS-iTBS/ts-DCS**  **(3 min)** | 8.14 (3.57) | 7.19 | 5.30~12.90 | 1.02 (0.03) | 1.01 | 1.00~1.05 | *1.67 (0.67)* | *1.47* | | *1.11~2.64* |
| **rTMS-iTBS/ts-iTBS**  **(3 min)** | 10.50 (5.85) | 10.20 | 4.80~16.80 | 0.99 (0.05) | 0.99 | 0.93~1.04 | 1.42 (0.94) | 1.18 | | 0.58~2.74 |
| **rTMS/ts-DCS**  **(20 min)** | *14.20 (5.64)* | *15.00* | *8.20~19.40* | *1.10 (0.14)* | *1.10* | *0.96~1.25* | 1.07 (0.46) | 0.93 | | 0.70~1.58 |
| **rTMS/ts-iTBS**  **(20 min)** | 10.16 (4.03) | 9.10 | 6.00~16.80 | 1.01 (0.04) | 1.02 | 0.96~1.05 | 1.28 (0.69) | 1.03 | | 0.87~2.51 |
| Group is a univariate factor; BBB scale, Basso, Beattie, and Bresnan; Pre_MEP, includes Pre_10min and Pre_5min MEP; Post_MEP, includes Post_5min, Post_10min, Post_15min, Post_20min, Post_25min, and Post_30min; Control, *n* = 4; Sham, *n* = 4; rTMS-iTBS/ts-DCS, *n* = 4; rTMS-iTBS/ts-iTBS, *n* = 4; rTMS/ts-DCS, *n* = 3; rTMS/ts-iTBS, *n* = 5. | | | | | | | | | | |
